# Physical Sensors Based on Lamb Wave Resonators

**DOI:** 10.3390/mi15101243

**Published:** 2024-10-09

**Authors:** Zixia Yu, Yongqing Yue, Zhaozhao Liang, Xiaolong Zhao, Fangpei Li, Wenbo Peng, Quanzhe Zhu, Yongning He

**Affiliations:** 1 The Key Lab of Micro-Nano Electronics and System Integration of Xi’an City, School of Microelectronics, Faculty of Electronic and Information Engineering, Xi’an Jiaotong University, Xi’an 710049, Chinawpeng33@mail.xjtu.edu.cn (W.P.);; 2 Shaanxi Advanced Semiconductor Technology Center Co., Ltd., Xi’an 710077, China

**Keywords:** Lamb wave, sensor, electrode configuration, sensitivity, piezoelectric effect

## Abstract

A Lamb wave is a guided wave that propagates within plate-like structures, with its vibration mode resulting from the coupling of a longitudinal wave and a shear vertical wave, which can be applied in sensors, filters, and frequency control devices. The working principle of Lamb wave sensors relies on the excitation and propagation of this guided wave within piezoelectric material. Lamb wave sensors exhibit significant advantages in various sensing applications due to their unique wave characteristics and design flexibility. Compared to traditional surface acoustic wave (SAW) and bulk acoustic wave (BAW) sensors, Lamb wave sensors can not only achieve higher frequencies and quality factors in smaller dimensions but also exhibit superior integration and multifunctionality. In this paper, we briefly introduce Lamb wave sensors, summarizing methods for enhancing their sensitivity through optimizing electrode configurations and adjusting piezoelectric thin plate structures. Furthermore, this paper systematically explores the development of Lamb wave sensors in various sensing applications and provides new insights into their future development.

## 1. Introduction

Acoustic wave devices include surface acoustic wave (SAW) resonators and bulk acoustic wave (BAW) resonators, which have significant advantages, such as high sensitivity [1], non-contact measurement [2], miniaturization, and fast response [3]. They are utilized in industrial monitoring, medical diagnostics, and communication technologies [4,5,6]. In 1973, Toda [7] first realized a Lamb wave device on a PZT ceramic plate. Subsequently, the A_0_ mode Lamb wave resonator (LWR) has been widely studied. However, due to the slower phase velocity of the A_0_ mode, its ability to rapidly radiate energy into surrounding liquids in liquid sensing applications is limited [8]. In contrast, the S_0_ mode has a higher phase velocity of approximately 10,600 m/s [9]. In 2005, the first AlN-based S_0_ mode LWR was developed [10]. Recently, Lamb wave resonators operating in the lowest order symmetric mode (S_0_) have been extensively explored.

The Lamb wave is an elastic wave that propagates along thin plates and is excited and detected through interdigital electrodes attached to a piezoelectric thin plate. An LWR consists of a piezoelectric thin plate and an interdigital transducer (IDT). Typically, the fabrication of Lamb wave devices involves depositing a piezoelectric thin film, such as AlN, ZnO, or GaN, on a Si substrate, followed by the deposition of metal to form electrodes on the piezoelectric thin film. Their compatibility with complementary metal oxide semiconductor (CMOS) processes makes LWRs convenient for integrated circuits and microsystems. Compared to SAW resonators, Lamb wave resonators exhibit a higher phase velocity [9] and a greater effective electromechanical coupling coefficient [11], enabling a wider bandwidth and higher frequency.

**Figure 1:**
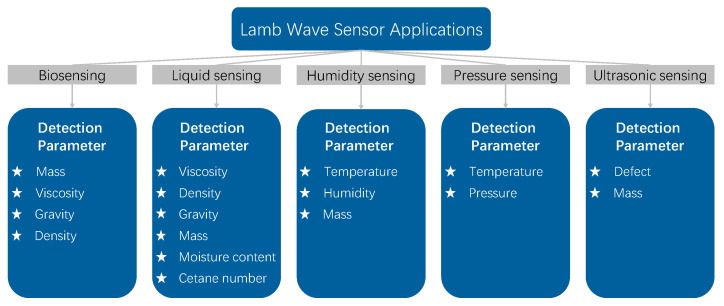
Classification of applications and detection parameters for LWRs as sensors.

LWRs have been exhibited in various fields, including biosensing [12,13,14], liquid sensing [15,16,17,18], humidity sensing [19,20,21], and pressure sensing [22,23,24]. In the field of biosensing, LWRs have been applied in the detection of DNA, bacteria, and biopharmaceuticals. In the field of liquid sensing, LWRs are capable of detecting parameters such as viscosity, density, gravity, and moisture content. LWRs can adjust frequency by modifying interdigital electrode size and piezoelectric materials, making them useful for ultrasonic devices [25,26] for steel plate defect detection. Additionally, LWRs have also been applied in the fields of strain sensing [27,28] and chemical sensing [29]. Figure 1 illustrates the application of LWRs as sensors in various fields.

In recent years, Lamb wave resonators have been used in the field of sensing, and there are relatively many theoretical studies. However, there is currently a lack of systematic summaries of these studies. In order to better promote the development of Lamb wave sensors, this paper aims to review the advancements of Lamb wave sensors over the past decade, outlining their fundamental structure, operating principles, and key parameters while exploring their sensing mechanisms and analyzing how material selection influences performance. By summarizing relevant research methodologies and findings, this paper seeks to provide a reference for the further advancement of this field.

## 2. Fundamental Principles of LWRs

### 2.1. Structure of the LWR

**Figure 2:**
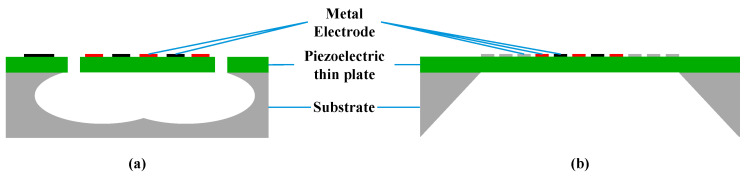
Two topologies of LWRs: (**a**) edge-type and (**b**) grating-type.

The LWR is composed of a piezoelectric thin plate and IDTs, combining the advantages of both BAW and SAW resonators, and has the characteristics of ultra-high phase velocity ($v_{p}$) and multi-frequency excitation [30]. Unlike the upper and lower electrode structures of traditional BAW resonators, LWRs rely on surface-interdigitated electrodes to generate resonance. Although in some designs, LWRs may use grating structures to guide or control acoustic waves, their designs are generally more flexible, typically relying on thin film structures and boundary conditions rather than depending on grating reflectors to confine acoustic waves and form resonance as in SAW resonators. In this regard, we present two different LWR topologies with distinct reflection mechanisms, namely, edge-type [31,32] and grating-type [33,34], as shown in Figure 2.

The edge-type LWR reflects waves via the suspended edge of a thin plate, while the structure of the grating-type LWR is similar to that of a SAW resonator, using a grating reflector with electrode widths of λ/4 to reflect. Unlike SAW resonators, in which wave propagation occurs on the surface, LWRs propagate waves within a suspended piezoelectric thin plate. The thickness of the piezoelectric thin plate can range from several hundred nanometers to a few micrometers, rather than hundreds of micrometers. Furthermore, by optimizing the design of edge-type reflectors, such as using damped edge reflectors [35], broadband spurious modes can be effectively suppressed.

**Figure 3:**
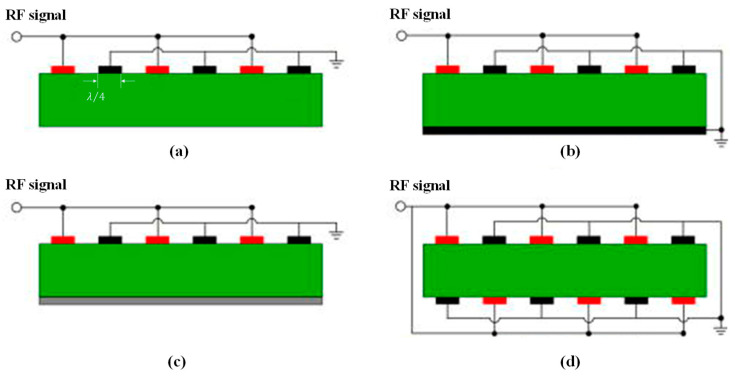
Four transducer configurations of single-port LWRs [36]: (**a**) single-IDT; (**b**) IDT/grounded-BE; (**c**) IDT/floating-BE; (**d**) double-IDT.

In order to enhance the performance of the resonator, various design approaches can be adopted for the interdigital electrode. Several common IDT configurations of single-port LWRs are illustrated in Figure 3, including the single-IDT electrode configuration, the IDT with a grounded bottom electrode (BE) configuration, the IDT with floating-BE configuration, and the double-IDT electrode configuration [36]. These different electrode configurations influence the effective electromechanical coupling coefficient ($k_{eff}^{2}$) of the resonator.

**Figure 4:**
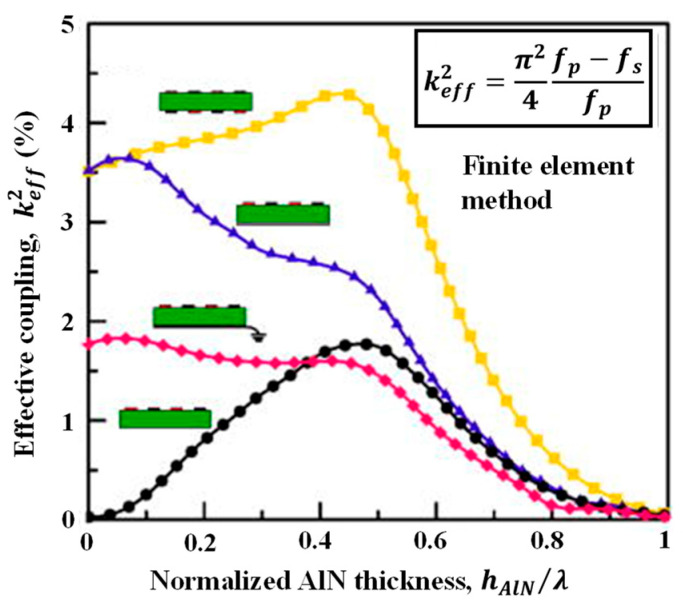
Effective electromechanical coupling coefficient of four transducer configurations in AlN thin plates of S_0_ mode [36], where $f_{p}$ is the parallel resonant frequency and $f_{s}$ is the series resonant frequency. The thickness of the piezoelectric thin plate affects the resonant frequency, which, in turn, influences the $k_{eff}^{2}$. Different colored lines represent different transducers, and their structures are shown in the figure.

Taking the LWR with AlN as the piezoelectric thin plate and Pt as the electrode as an example [36], the curve of the $k_{eff}^{2}$ is illustrated in Figure 4. The double-IDT configuration enhances the coupling of the electroacoustic field by forming a complete electric field loop within the thin plate, thereby improving the energy conversion efficiency and achieving a higher $k_{eff}^{2}$. In contrast, the $k_{eff}^{2}$ of the IDT with the floating-BE configuration is higher than that of the IDT with the grounded-BE configuration because the latter has a larger static capacitance in the LWR, and its unidirectional vertical electric field results in a lower $k_{eff}^{2}$. However, the single-IDT electrode configuration only forms an electric field at the top, leading to reduced electroacoustic coupling efficiency, thus resulting in the lowest $k_{eff}^{2}$. To achieve a higher $k_{eff}^{2}$, the double-IDT electrode configuration is a priority, with its maximum achievable $k_{eff}^{2}$ approaching 4.5%.

In fact, in addition to the electrode configuration on the electromechanical coupling coefficient, the fabrication process is crucial for determining LWR performance. LWR technology is similar to film bulk acoustic resonator (FBAR) technology, both of which are realized through micromachining on a piezoelectric thin plate. For the edge-type LWR, as illustrated in Figure 2a, the suspended piezoelectric thin plate edges are formed by etching using photoresist or SiO_2_ [37] as a hard mask, ensuring vertical edges and high quality. The release of the piezoelectric thin plate can be achieved through dry etching methods using XeF₂ [37,38] or SF₆ [39] or through the wet etching processes. For the grating-type LWR, as illustrated in Figure 2b, the standard three-step dry etching method Bosch process is typically used to etch the Si substrate from the backside, thereby isolating the device from the support layer.

Upon excitation of the device, the Lamb wave propagates along both sides of the x-axis. When the wave reaches the boundary of the resonator, reflection occurs. The reflected wave superimposes with the incident wave to form a standing wave, thereby inducing resonance. Whether the LWR is of the grating type or edge type, its resonant frequency is determined by both the phase velocity of the wave and the width of the interdigital electrodes, as follows [40]:

(1) $$f_{s}=\frac{v_{p}}{\lambda }=\frac{v_{p}}{2p}$$

where $f_{s}$ is the series resonant frequency, $v_{p}$ is the phase velocity, λ is the wavelength, and p is the pitch, which represents the spacing between the interdigital electrodes.

### 2.2. Calculation Methods for Lamb Wave Modes

**Figure 5:**
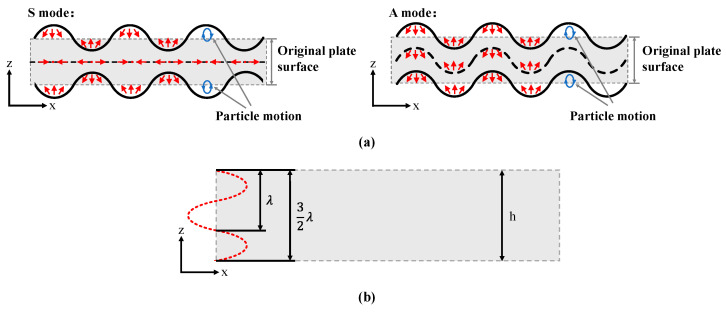
(**a**) Two modes of the Lamb wave; (**b**) schematic diagram of a finite-length thin plate.

In thin plate materials, there are two types of plate waves, the Lamb wave and the shear horizontal (SH) wave [41]. The Lamb wave is formed by the coupling of a longitudinal (L) wave and a shear vertical (SV) wave, inducing displacements in the x and z directions. The Lamb wave can be categorized into a symmetric mode (S mode) and an antisymmetric mode (A mode) based on particle motion characteristics, as illustrated in Figure 5a. From the figure, it can be observed that the vibration patterns of the S mode Lamb wave are symmetric in the upper and lower thin plate surfaces, with particles on the upper and lower surfaces having identical displacements in the x direction but opposite displacements in the z direction, resulting in elliptical particle trajectories. In contrast, the A mode Lamb wave exhibits identical vibration patterns in the upper and lower thin plate surfaces, with particles having the same displacement components in both the x and z directions, resulting in periodic elliptical motion. Based on the standing wave number n in the thickness direction of the thin plate, the nth-order symmetric Lamb wave is denoted as S_n_, and the nth-order antisymmetric Lamb wave is denoted as A_n_. The mode order *n* is determined by the relationship between the half-wavelength (λ/2) of the standing wave and the thin plate thickness. Figure 5b illustrates the schematic of the finite-length thin plate, in which the relationship between the mode order n and the thin plate thickness h is given as follows:

(2) $$h=\frac{\lambda }{2}\cdot n\;\left(n=0,1,2,\cdots \right)$$

This equation can be used to determine the mode order, but the mode identification requires judgment based on its characteristics. According to the definition of the Lamb wave, although it differs from the SAW, the Lamb wave shares many similarities with the Rayleigh wave. When a Rayleigh wave propagates through a sufficiently thin plate (h<10λ), it can transform into a Lamb wave. Typically, these two types of waves can be distinguished by the ratio of the wavelength λ to the thin plate thickness h. If λ≪h, it is classified as a Rayleigh wave; if λ≫h, it is classified as a Lamb wave; and if λ/h≈1, it represents a transition mode between the two [42].

### 2.3. Dispersion Characteristics of the Lamb Wave

The symmetric and antisymmetric modes of the Lamb wave satisfy the Rayleigh–Lamb (R-L) frequency equations [43], exhibiting dispersion characteristics. Their phase velocity is related to frequency and wavelength. The specific dispersion relation can be expressed as follows [44]:

(3) $$S_{n}:\;\frac{tan\left(\frac{\beta h}{2}\right)}{tan\left(\frac{\alpha h}{2}\right)}=-\frac{4\alpha \beta k^{2}}{\left(k^{2}-\beta^{2}\right)}$$

(4) $$A_{n}:\;\frac{tan\left(\frac{\beta h}{2}\right)}{tan\left(\frac{\alpha h}{2}\right)}=-\frac{\left(k^{2}-\beta^{2}\right)}{4\alpha \beta k^{2}}$$

where $\alpha^{2}=\frac{\omega^{2}}{\nu_{l}^{2}}-k^{2}$, $\beta^{2}=\frac{\omega^{2}}{\nu_{t}^{2}}-k^{2}$, h is the thickness of the piezoelectric thin plate, ω is the angular frequency, k is the wave number, $v_{l}$ is the longitudinal wave velocity of the isotropic material, and $v_{t}$ is the transverse wave velocity of the isotropic material.

Jie Zou et al. [45] analyzed the first eight Lamb wave modes in the AlN plate using the finite element method (FEM) and Adler’s matrix. The analysis includes phase velocity, dispersion curves, group velocity, and the frequency–temperature coefficient (TCF). They employ normalized frequency and normalized wavenumber, which helps to more clearly reflect the material’s dispersion characteristics. The cutoff frequencies of higher-order modes (the frequencies at k=0) are higher than those of lower-order modes (where the cutoff frequency is 0). The phase velocity of acoustic waves can be calculated using the following equation:

(5) $$v_{p}=\frac{\omega }{k}$$

where ω is the angular frequency.

As the AlN plate thickness varies, the phase velocity of the S_0_ mode changes gently, while that of the A_0_ mode increases with thickness. In contrast, the phase velocity of higher-order modes decreases as the AlN plate thickness increases. Moreover, the energy propagation velocity, also known as the group velocity, also reflects the propagation behavior in different modes. The group velocity can be expressed by differentiating the dispersion curve, as follows:

(6) $$v_{g}=\frac{\partial \omega }{\partial k}$$

When the wave number is zero, the group velocity of higher-order vibration modes is zero, indicating that energy will not leak along the x direction of the IDT. In contrast, lower-order modes (such as the A_0_ mode and S_0_ mode) are generated by the electrical excitation applied by the IDT, with their wave number determined by the IDT’s period; therefore, their group velocity is non-zero. In this case, grating reflectors or suspended edges can be added on both sides of the IDT to confine the acoustic energy propagating within the IDT region, thereby preventing energy leakage and enhancing the energy transmission efficiency of the LWR.

According to Equation (7) [46], the TCF is closely related to the temperature dependence of the phase velocity and the thermal expansion coefficient in the direction of acoustic wave propagation. Therefore, temperature variations can affect the dispersive phase velocity of acoustic waves, subsequently influencing the operating frequency of the resonator.

(7) $$TCF=\frac{1}{f}\frac{\partial f}{\partial T}=\frac{1}{v_{p}}\frac{\partial v_{p}}{\partial T}-\frac{1}{\lambda }\frac{\partial \lambda }{\partial T}=\frac{1}{v_{p}}\frac{\partial v_{p}}{\partial T}-\alpha_{z}$$

where $\alpha_{z}$ corresponds to the thermal expansion in the thickness direction of the substrate.

For multiple Lamb wave modes of the AIN thin plate, except for the S_0_ and S_1_ modes, the first-order TCFs of the other Lamb wave modes are all approximately −25 ppm/°C. Notably, the S_0_ and S_1_ modes exhibit entirely different temperature dependencies. The TCF of the S_0_ mode shows a greater variation with h_AlN_/λ, which is attributed to its strong correlation with the transverse field. Since the elastic modulus of the transverse field is less sensitive to temperature changes, the S_0_ mode exhibits the optimal TCF. In contrast, the S_1_ mode involves more vertical vibration, with an elastic modulus that is more sensitive to temperature changes, thereby resulting in the worst TCF. Considering that the TCF of AlN is negative, to achieve a zero TCF, temperature compensation can be realized by introducing materials with a positive TCF, such as SiO_2_ [47] or BN [48].

In summary, higher-order Lamb wave modes exhibit higher phase velocities and are suitable for high-frequency applications. However, the strong dispersion characteristics of higher-order modes impose greater demands on the fabrication process. Currently, most research is primarily focused on the S_0_ mode of the AlN thin plate due to its low phase velocity dispersion, allowing resonators to operate over a wide frequency range and enabling the realization of zero TCF devices [40], thereby ensuring stable device performance. Additionally, researchers are exploring the A_1_ and S_1_ modes of Lamb wave, which demonstrate higher acoustic velocities and larger effective electromechanical coupling coefficients, making them suitable for high-frequency acoustic devices [49].

### 2.4. Key Parameters of Lamb Wave Resonator

In the performance evaluation of LWRs, the quality factor (Q factor) and the effective electromechanical coupling coefficient are commonly used to measure energy conversion efficiency [50].

#### 2.4.1. Quality Factor

The quality factor represents the ratio of the energy received to the energy dissipated by a resonator during one period of oscillation. A higher Q factor contributes to reduced insertion loss, improved sensor resolution, and enhanced oscillator stability [51,52]. In practical applications, it is often difficult to directly calculate the Q factor of a resonator through its definition. Typically, the Q factor is calculated at the resonant point by the ratio of the resonant frequency to the 3 dB bandwidth [53], as shown in Equation (8).

However, this method is only applicable near the resonant frequency and cannot evaluate the resonator’s performance across the entire frequency range. To address this problem, the Bode Q equation [54] can be used, as shown in Equation (9). This method allows for the calculation of the Q factor within a specific frequency band, which can more accurately reflect the relationship between the Q factor and the resonant frequency.

(8) $$Q_{s}=\frac{f_{s}}{∆f_{3dB}}$$

(9) $$Q\left(f\right)=\frac{2\pi f\tau \left(f\right)\left|S_{11}\right|}{1-{\left|S_{11}\right|}^{2}}$$

where $∆f_{3dB}$ is the 3dB bandwidth, and $\tau \left(f\right)$ is the group delay of S_11_.

Existing research indicates that altering the geometry of piezoelectric thin plates, such as using butterfly-shaped thin plates [55] and chamfered corner thin plates [56], can effectively suppress tether displacement and reduce anchor loss. Additionally, increasing the number of tethers [57] can effectively suppress parasitic modes. The traditional method of using tethers for anchoring is common but can be replaced by a planar ring-shaped phononic crystals (PnCs) matrix [58], which enhances structural robustness and improves resistance to mechanical shock. Furthermore, modifying the IDT shape [59] also contributes to the improvement of the resonator’s Q factor.

#### 2.4.2. Effective Electromechanical Coupling Coefficient

The $k_{eff}^{2}$ reflects the efficiency of conversion between mechanical and electrical energy forms of the resonator. The larger $k_{eff}^{2}$, the higher conversion efficiency. When the resonator is used in a sensor, $k_{eff}^{2}$ will influence the sensitivity of the sensor. Typically, $k_{eff}^{2}$ is calculated using the series resonant frequency$\;f_{s}$ and the parallel resonant frequency $\;f_{p}$ of the resonator, as follows [60]:

(10) $$k_{eff}^{2}=\frac{\pi^{2}}{4}\frac{\left(f_{p}-f_{s}\right)}{f_{p}}$$

Furthermore, the $k_{eff}^{2}$ is also influenced by electrode configuration, electrode materials, piezoelectric materials, piezoelectric thin plate thickness, and Lamb wave modes [36,45]. Jie Zou et al. [36] conducted an analysis of LWR based on AlN thin films and simulated the $k_{eff}^{2}$ of four different electrode configurations in the S_0_ mode with varying AlN thicknesses. The results indicated that under the same material and thickness ratio conditions, the double-IDT electrode configuration consistently exhibited the highest $k_{eff}^{2}$, as shown in Figure 4.

In practical design, in addition to the electrode configuration, materials, and mode selection, other resonator parameters must also be considered to achieve optimal performance. For a detailed analysis and discussion of the $k_{eff}^{2}$ of AlN LWRs, please refer to references [36,45].

## 3. Manufacturing Materials for Sensors

### 3.1. Piezoelectric Materials

Currently, the piezoelectric materials used in LWRs primarily include quartz, LiNbO_3_, AlN, ZnO, and GaN. Among these, quartz is an insulating material, while LiNbO_3_, AlN, ZnO, and GaN are wide-bandgap semiconductor materials. Due to its high frequency stability, strong corrosion resistance, and low aging rate, quartz has been widely used in the field of oscillator fabrication [61]. Although LWRs based on quartz exhibit a high Q factor [62], their application is somewhat limited because quartz cannot be fully integrated with CMOS technology [63]. ZnO is a commonly used piezoelectric material in acoustic wave devices, such as BAW resonators [64] and SAW resonators [65]. However, ZnO tends to form oxygen vacancies during the fabrication process, and the rapid diffusion of Zn ions may lead to contamination issues, thereby affecting device performance. In contrast, AlN with its high acoustic velocity, as well as LiNbO_3_, with its high dielectric constant and high effective electromechanical coupling coefficient, have become mainstream materials for fabricating LWRs. Although the effective electromechanical coupling coefficient of GaN is much lower than that of other materials, its remarkable electron mobility has made it a research focus, with related studies already being reported [66]. Table 1 provides a comparison of the piezoelectric materials currently used in the development of Lamb wave sensors.

### 3.2. Electrode Materials

In addition to the influence of piezoelectric materials on resonator performance, the selection of electrode materials also significantly impacts the resonator’s performance. An ideal electrode material should possess high electrical conductivity, process compatibility, thermal stability, durability, and corrosion resistance. Moreover, the electrode material should match the lattice of the piezoelectric material to reduce lattice distortion, thereby optimizing the quality of the piezoelectric thin plate; alternatively, it should have a significant difference in acoustic impedance with the piezoelectric material to enhance the $k_{eff}^{2}$.

Recently, extensive studies have explored the effects of various electrode materials (such as Al [80,81], Pt [82], Au [83], and Mo [84]) on the performance of LWRs. Jie Zou et al. [85] systematically summarized the role of electrode materials in AlN LWRs. However, in sensor applications, the selection of electrode materials may significantly affect the sensor’s sensitivity.

Based on Table 1 and Table 2, it can be concluded that the greater the difference in acoustic impedance between the electrode materials (such as Au, Mo, and W) and the piezoelectric materials, the more significantly the effective electromechanical coupling coefficient of the resonator increases. Additionally, high-conductivity materials (such as Al, Au, and Ag) can effectively reduce resistive losses, thereby improving the signal–noise ratio (SNR) of the sensor and enhancing its sensitivity. Therefore, the selection of electrode material has a direct impact on the sensor’s performance.

**Figure 6:**
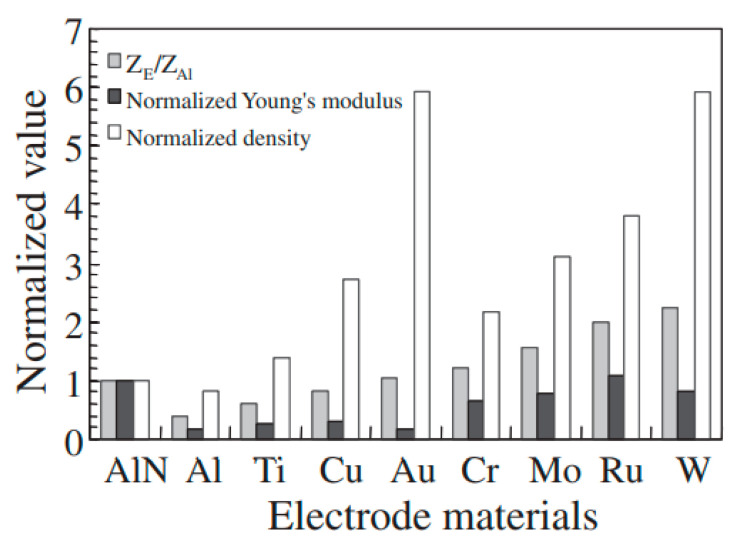
Comparison of acoustic impedance, Young’s modulus, and density for different electrode materials [88]. All parameter values are normalized relative to the characteristics of AlN.

When LWRs are used as sensors, different types of sensors have different requirements for electrode materials. For liquid sensors, the electrode materials must exhibit good chemical stability and acoustic properties to minimize the reflection of acoustic waves between the liquid and the electrodes. In liquid sensor applications [16,17], AlN is commonly used as the piezoelectric material, while Al is used as the electrode material. This is because AlN exhibits excellent piezoelectric properties and a high acoustic velocity, which contribute to enhancing the sensitivity and resolution of the sensors. Additionally, Al offers good conductivity and a relatively matching thermal expansion coefficient with AlN, helping to reduce the mismatch caused by thermal stress. However, Al has poor chemical stability, making it prone to corrosion in liquid environments. Its relatively low Young’s modulus (as shown in Figure 6) also makes it susceptible to deformation under high-stress conditions, and it is prone to failure due to thermal stress in environments with large temperature fluctuations. Therefore, current research trends to use Lamb wave devices fabricated with Mo [86,87], with a high Young’s modulus (as shown in Figure 6) and excellent corrosion resistance, or Au/Ti [16], with high conductivity and chemical stability, for liquid sensor applications.

For biosensors, the electrode materials should possess biocompatibility and the capability for chemical functionalization to ensure effective operation in biological environments. ZnO and Al are commonly used as piezoelectric and electrode materials [13,14], respectively, as ZnO possesses a certain degree of biocompatibility and good chemical stability, which enhances the sensor’s response to biological signals. Additionally, Al as an electrode material provides good conductivity and thermal stability, contributing to enhancing the performance of the sensor. However, due to its relatively poor chemical stability, Al is typically used as the bottom electrode of the resonator, while the top electrode can be made of Au, which exhibits high chemical stability, strong corrosion resistance, and high biocompatibility.

For pressure sensors, the electrode materials need to have sufficient mechanical strength and thermal stability to maintain reliability and stability under varying pressure conditions. Early Lamb wave pressure sensors predominantly utilized AlN as the piezoelectric material and Al as the electrode material [22,46]. The piezoelectric properties of AlN enable it to effectively convert mechanical pressure into electrical signals, thereby improving the sensitivity of the sensor. Currently, similar to the field of liquid sensing, the use of Mo [23] as an electrode material further improves the reliability and stability of pressure sensors.

**Table 2:** Parameters of commonly used electrode materials [36,76,89,90,91,92,93].

Material	Al	Au	Mo	Ag	W	Ti
$\mathrm{Longitudinal}\;\mathrm{wave}\;\mathrm{acoustic}\;\mathrm{velocity}/m\cdot s^{-1}$	6418	3200	6300	3600	5211	6072
$\mathrm{Density}/g\cdot {\mathrm{cm}}^{-3}$	2.7	19.4	10	10.5	19.3	4.5
$\mathrm{Acoustic}\;\mathrm{impedance}\;(10^{6}\;\mathrm{kg}\cdot (m^{-2}s^{-1})$)	17.3	61.8	63	38.2	100.6	27.3
Conductivity (MS/m)	36.9	44.2	18.7	62.1	18	2.34
Young’s modulus (GPa)	70	79	316.5	82.5	332	115

## 4. LWR as Sensors

Currently, LWRs exhibit extremely high sensitivity to environmental changes due to their high Q factor and frequency stability. Their multi-mode characteristics not only enable multifunctionality in sensor applications but also optimize the overall performance. In the following sections, we will summarize the applications of Lamb wave sensors in various fields, including biosensing, liquid detection, pressure sensing, and humidity measurement.

### 4.1. LWR Biosensors

**Figure 7:**
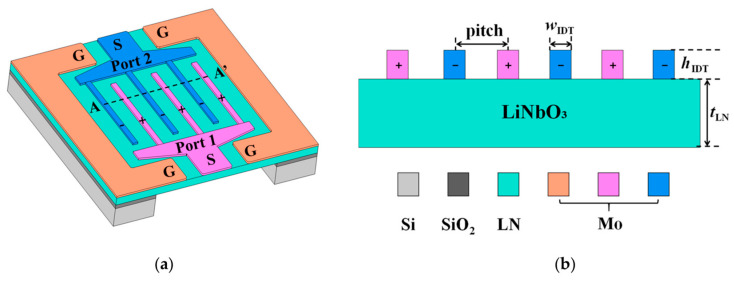
Common LWR biosensors [12]: (**a**) structural design; (**b**) cross-sectional diagram.

In recent years, biosensors based on Lamb wave technology have been widely used. Researchers are particularly focused on enhancing the sensitivity and detection limits of biosensors to improve their ability to detect substances such as bacteria, fungi, and proteins. Figure 7 illustrates a schematic of the commonly used Lamb wave biosensors, in which a layer of polymethyl methacrylate (PMMA) on the surface of the resonator is employed for the mass sensing of biomolecules.

**Figure 8:**
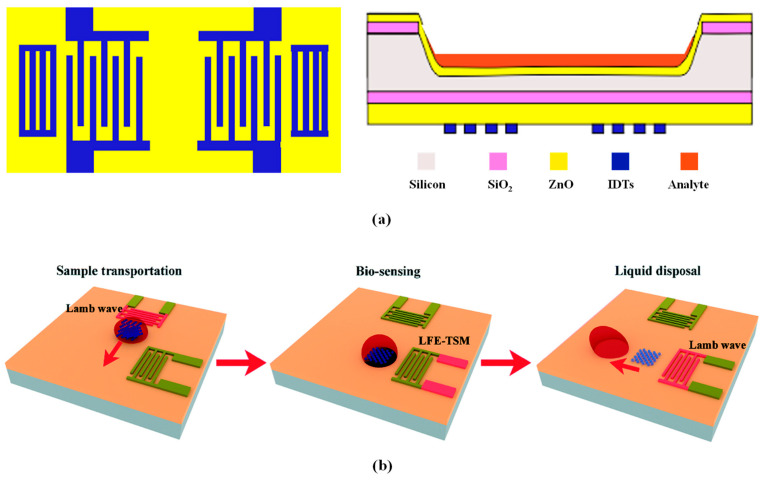
Applications of LWR biosensors: (**a**) electrode structure and model diagram of an inverted LWR biosensor based on ZnO/SiO_2_/Si/ZnO film [13]; (**b**) schematic diagram of a flexible acoustic sensor for biosensing based on LFE-TSM/Lamb wave hybrid mode [14].

Typically, the LWR acts as a biosensor by binding target biomolecules to recognition molecules fixed on the resonator surface, causing a change in the mass of the LWR and thereby resulting in a shift in resonant frequency. By monitoring changes in the resonant frequency, qualitative and quantitative analyses of the target biomolecules can be conducted. Recently, some studies have developed various Lamb wave sensors for different biological applications by employing inverted structures and acoustic wave integration, as illustrated in Figure 8. Manisha Bharati et al. [13] proposed a LWR biosensor based on an inverted structure for detecting Neisseria meningitidis, which causes bacterial meningitis. This sensor utilizes the high isoelectric point of the ZnO film to effectively bind DNA at neutral pH. Additionally, the inverted structure design effectively prevents short-circuiting of the IDT due to liquid samples and provides a sufficiently large surface area to facilitate the binding of analytes to the biological receptors. This design enables the sensor to exhibit high sensitivity and low detection limits in both the A_0_ and S_0_ modes. Ran Tao et al. [14] integrated the Lamb wave, which is suitable for fluidic actuation applications, and the thickness shear wave for biosensing into a flexible device, enabling the transport and handling of liquid volumes and the detection of the chemotherapeutic drug imatinib. Hongxiang Zhang et al. [94] designed a biosensor comprising four LWRs that locally enriched biological particles in the liquid through vortices induced by the LWR array. The acoustic streaming effects of the LWR in liquid environments were investigated through theoretical analysis and finite element numerical simulations, and experimental validation demonstrated the capability to effectively capture biological particles in a 1 μL droplet. Table 3 summarizes the characteristics of several LWR biosensors reported by researchers.

In biological mass sensing, PMMA is commonly used as a sensing layer to adsorb target biomolecules in liquids.

**Figure 9:**
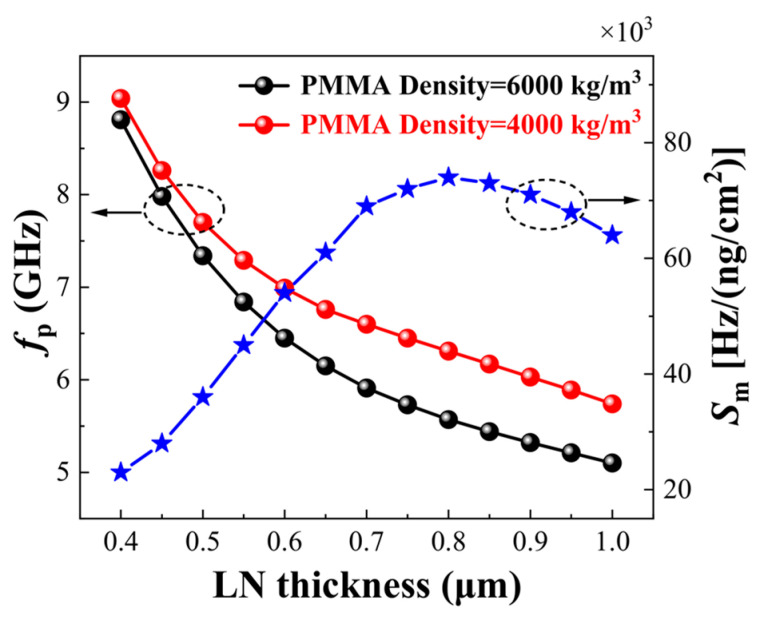
Curves showing the influence of piezoelectric film thickness on sensor sensitivity [12].

Typically, the mass sensitivity of Lamb wave biological mass sensors is related to the thickness of the piezoelectric thin plate, as shown in Figure 9. The mass sensitivity of the sensor can be optimized by adjusting the plate thickness. In fact, the thickness of the piezoelectric layer affects the mass sensitivity of the biological mass sensor while the thickness and density of the sensing layer, along with the electrode thickness, also influence the mass sensitivity of the biological mass sensor. According to Equation (11) [12], the mass sensitivity $S_{m}$ is negatively correlated with the change in the thickness and density of the sensing layer. However, by keeping the thickness and density of the sensing layer constant, increasing the thickness of the electrode can enlarge the surface area of the sensing layer, thereby enhancing its mass sensitivity [95]. It is important to note that excessively increasing the thickness of the electrode or the sensing layer may introduce excessive damping and energy loss, which can ultimately reduce the mass sensitivity.

(11) $$S_{m}=\left|\frac{∆f}{T_{s}∆\rho_{s}}\right|$$

where ∆f is the frequency offset under different gravitational loads, and $T_{s}$ and $∆\rho_{s}$ are the changes in the thickness and density of the sensing layer, respectively.

### 4.2. LWR Liquid Sensors

Sensors based on Lamb wave technology are not only used for biosensing but also for detection in liquid media. Currently, most researchers employ LWRs with S_0_ mode and A_0_ mode to achieve liquid sensing. The S_0_ mode experiences minimal attenuation in liquids, with the primary source of attenuation being the friction between the surface of the piezoelectric thin plate and the liquid. In contrast, the A_0_ mode, due to its bending vibration characteristics, exhibits three main sources of attenuation, resulting in more significant attenuation. These characteristics make them suitable for different liquid sensing applications [96].

The working principle of the Lamb wave liquid sensor can be described by Equation (12) [97]. When liquid is dripped onto the sensor surface, the resonant frequency changes, with the frequency offset primarily determined by the density and viscosity of the liquid.

(12) $$∆f=-f_{s}^{3/2}\sqrt{\frac{\rho_{l}\eta_{l}}{\rho_{0}\eta_{0}\pi }}$$

where ∆f is the frequency offset induced by the liquid, $f_{s}$ is the series resonant frequency of the liquid sensor, and $\rho_{l}$ and $\eta_{l}$ are the density and viscosity of the liquid, respectively, while $\rho_{0}$ and $\eta_{0}$ are the density and viscosity of the piezoelectric thin plate, respectively.

**Figure 10:**
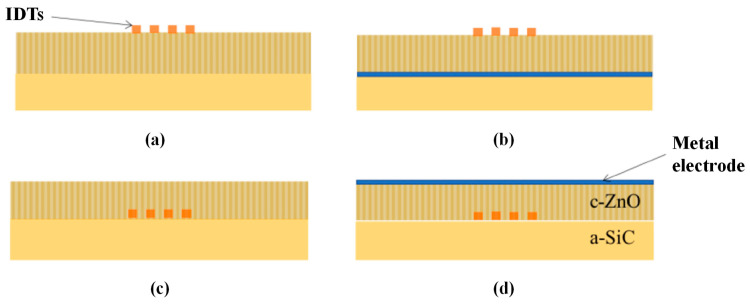
Four coupling configurations of LWR liquid sensors [100]: (**a**) sfT; (**b**) smfT; (**c**) sTf; (**d**) sTfm.

The Lamb wave sensor employs the S_0_ mode and an open-bottom topology (as illustrated in Figure 10a), which is suitable for measuring the dielectric constant of liquids. Due to the low attenuation of the S_0_ mode in liquids, when the dielectric constant of the liquid increases, electrical energy can still be effectively stored within the resonant cavity, thereby enhancing the effective electromechanical coupling coefficient [98]. To further enhance the effective electromechanical coupling coefficient, researchers have explored four electroacoustic coupling configurations by varying the position of the IDT. These configurations include placing the IDT on the surface of the film (sfT) at the substrate/film interface (sTf) and adding a floating metal electrode opposite the IDT in the two aforementioned configurations (smfT and sTfm) [99], as illustrated in Figure 10.

**Figure 11:**
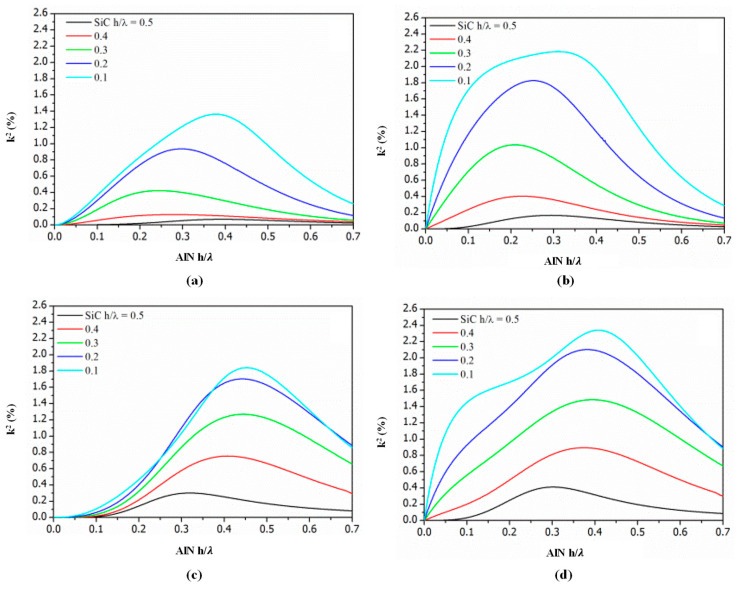
Curves of effective electromechanical coupling coefficients for four coupling configurations on c-AlN/SiC (001) <100> substrates [100]: (**a**) sfT; (**b**) smfT; (**c**) sTf; (**d**) sTfm.

Based on current research [99,100,101,102], regardless of whether ZnO or AlN are used and regardless of variations in the thickness-to-wavelength ratio (h/λ), both sTfm and smfT structures exhibit high effective electromechanical coupling coefficients. However, increasing the thickness of the SiC layer will reduce the electromechanical coupling coefficients. For instance, Figure 11 illustrates the k² dispersion curves for four coupling configurations in c-AlN/SiC(001)<100>.

Currently, research on Lamb wave liquid sensors primarily focuses on detecting parameters such as liquid density, viscosity, and dielectric constant. Table 4 summarizes several characteristics of Lamb wave liquid sensors reported by researchers.

**Figure 12:**
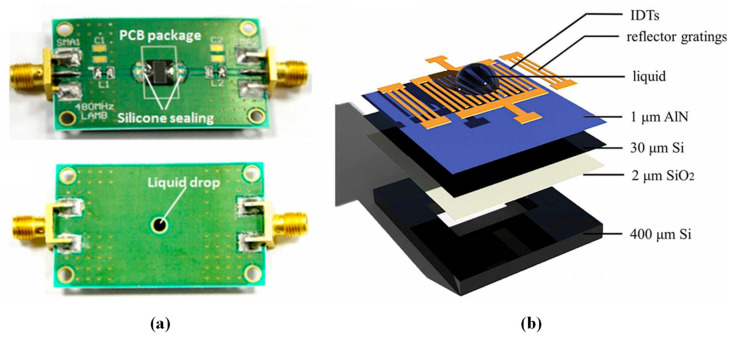
Applications of LWR liquid sensors: (**a**) model and physical diagram of a density and viscosity decoupled AlN Lamb wave sensor [16]; (**b**) two-dimensional array model broken view of a Lamb wave viscosity sensor [15].

Teona Mirea et al. [17,18,98] conducted finite element simulation analysis and experimental studies on S_0_ mode LWR, exploring the effects of liquid density, viscosity, and dielectric constant on the performance of the resonator. They found that the frequency offset is linearly related to the square root of the product of density and viscosity. Additionally, they detected changes in the dielectric constant by monitoring variations in the electromechanical coupling coefficient. Alexandros K. Pantazis et al. [103] experimentally tested S_0_ mode LWRs at different frequencies and found that the S_0_ mode LWR is more suitable for viscosity measurement when the operating frequency is below 200 MHz. However, due to the leakage of the S_0_ mode Lamb wave into the adjacent liquid layer [96], this sensor cannot measure density and viscosity separately. Tao Wang et al. [16] developed an AlN-based Lamb wave sensor that decouples density and viscosity (as shown in Figure 12a) and discovered the following two modes of particle motion on the backside: one parallel and the other perpendicular to the plate surface, with the frequency response determined by both viscosity and density. Qiong Liu et al. [15] proposed a two-dimensional array model for viscosity sensing (as shown in Figure 12b) and conducted experiments on liquid position, thickness, and viscosity. The results demonstrated that frequency offset is not only linearly related to the square root of viscosity but is also influenced by the liquid’s position and thickness within the sensor. To address the issue of acoustic radiation from LWRs in liquids, the propagation speed of the Lamb wave can be reduced by decreasing the thickness of the piezoelectric thin plate, but this affects the performance of Lamb wave liquid sensors. To this end, Feng Gao et al. [95] proposed a novel LWR utilizing high aspect ratio electrode (HARE) technology to achieve complete suppression of acoustic radiation in water, thereby enhancing the sensitivity and resolution of the sensor.

### 4.3. LWR Pressure Sensors

In recent years, due to their high sensitivity, low loss, and excellent stability in extreme environments, LWRs have shown very promising application prospects in harsh conditions. The working mechanism of Lamb wave pressure sensors is based on the influence of pressure on the propagation path of the Lamb wave, thereby altering its phase velocity and wavelength. In practice, it is common to detect pressure changes by monitoring the resonant frequency of the device. According to Equation (1), the series resonant frequency is determined by the phase velocity and wavelength of the Lamb wave, while the phase velocity in the piezoelectric material is related to Young’s modulus and the mass density of the material.

Taking AlN as an example, the elastic coefficient of this material is closely related to temperature or strain/stress. Consequently, changes in phase velocity caused by pressure or temperature can lead to a shift in the resonant frequency, as described by the following expression [46]:

(13) $$\frac{∆v_{p}}{v_{p}}=\frac{∆E}{2E}-\frac{∆\rho }{2\rho }$$

Thus, the offset of the resonant frequency can be expressed as follows [46]:

(14) $$\frac{∆f_{s}}{f_{s}}=\frac{∆v_{p}}{v_{p}}-\frac{∆\lambda }{\lambda }=\frac{\Delta E}{2E}-\frac{\Delta \rho }{2\rho }-\frac{∆\lambda }{\lambda }$$

where E is the Young’s modulus, ρ is the material density, and $f_{s}$ is the series resonant frequency of the pressure sensor.

Since Young’s modulus is composed of the elastic coefficient, it is closely related to temperature and pressure. The temperature dependence and pressure dependence of the Young’s modulus are expressed as follows [46]:

(15) $$E\left(T\right)=E\left(T_{0}\right)\left(1+TCE\Delta T+TCE2\Delta T^{2}\right)$$

(16) $$E\left(P\right)=E\left(P_{0}\right)\left(1+PCE\Delta T\right)$$

where $T_{0}$ is the reference temperature, Δ*T* is the temperature change, TCE and TCE2 are the first-order and second-order temperature coefficients, respectively, $P_{0}$ is the reference pressure, ΔP is the pressure change, and PCE is the first-order pressure coefficient.

Additionally, the pressure dependence of the resonant frequency is given as follows [46]:

(17) $$PCF=\frac{1}{f_{s}}\frac{\partial f_{s}}{\partial T}=\frac{1}{v_{p}}\frac{\partial v_{p}}{\partial P}-\frac{1}{\lambda }\frac{\partial \lambda }{\partial P}$$

The temperature dependence of material density *ρ* is expressed as follows [46]:

(18) $$\rho \left(T\right)=\rho \left(T_{0}\right)\left(1-\left(\alpha_{11}+\alpha_{22}+\alpha_{33}\right)∆T\right)$$

where $\alpha_{11}$, $\alpha_{22}$, and $\alpha_{33}$ correspond to the thermal expansion coefficients along different crystal orientations.

According to Equation (19) [46], thermal expansion can cause changes in the structural dimensions of AlN material, thereby affecting the wavelength.

(19) $$\lambda \left(T\right)=\lambda \left(T_{0}\right)\left(1+\alpha_{11}∆T\right)$$

The temperature dependence of the resonant frequency can be represented by Equation (7) [46], and its relationship with TCE and the thermal expansion coefficient is as follows:

(20) $$TCF=\frac{1}{2}\left(TCE-\alpha_{11}+\alpha_{22}+\alpha_{33}\right)+TCE2∆T$$

Currently, research primarily focuses on the TCF and the frequency pressure coefficient (PCF), as the measurement and compensation of these two parameters are crucial for achieving high-precision pressure sensors. Particularly in environments with significant temperature fluctuations, the stability of TCF and PCF directly impacts the performance of the sensor. With the continuous development of pressure sensor technology aimed at improving the performance of sensors to meet various application requirements, Table 5 presents several characteristics of Lamb wave pressure sensors reported by researchers.

**Figure 13:**
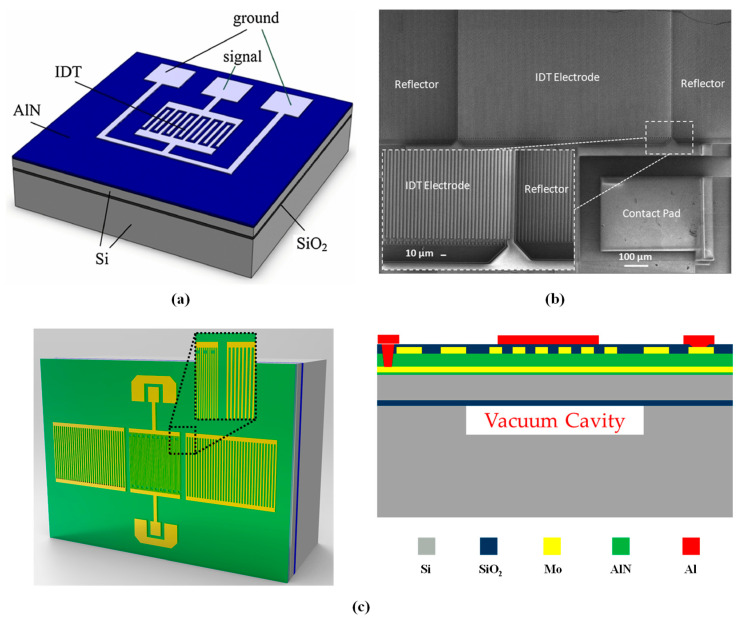
Applications of LWR pressure sensors: (**a**) lateral field excited (LFE) Lamb wave resonator for high-temperature pressure sensing [46]; (**b**) structural diagram of a piezoelectric sensor based on dual modes (LFE Lamb wave mode and SAW mode) [22]; (**c**) 3D structure diagram and cross-sectional of the dual-temperature-compensated Lamb wave pressure sensor [23].

P. Kropelnicki et al. [47] investigated the first AlN high-temperature pressure sensor based on the lateral field excited (LFE) Lamb wave mode, with its three-dimensional structure illustrated in Figure 13a. The device is composed of an AlN layer, an Si layer, and a buried silicon dioxide layer, effectively addressing the performance degradation of quartz pressure sensors at elevated temperatures. Xiaojing Mu et al. [23] designed a pressure sensor that incorporates both the LFE Lamb wave mode and the SAW mode, as illustrated in the overhead view in Figure 13b. This device features a grating-type Lamb wave resonator structure, with reflectors positioned at both ends of the IDT electrodes to enhance the excitation and detection effectiveness of acoustic waves. Additionally, based on the characteristic that the SAW and Lamb modes exhibit the same temperature behavior but different pressure behavior, a temperature compensation method was designed in the readout circuit. By using an external oscillator circuit to collect the resonant frequencies of the SAW and Lamb modes at room temperature, and according to the definition of beat frequency (Equation (21)) [22], the resonant frequencies of both modes can be input into subtractor and multiplier circuits to obtain the beat frequency.

(21) $$∆f=f_{Lamb}-\frac{f_{Lamb}}{f_{SAW}}\cdot f_{SAW}$$

Junwei Gu et al. [104] described a pressure sensor with multi-resonant modes based on LiNbO_3_ material. They established a three-dimensional resonator model through finite element analysis, thoroughly characterizing various resonant modes to enhance pressure sensitivity and temperature stability. This sensor also incorporates a thick SiO_2_ layer as a temperature compensation layer, thereby significantly reducing temperature drift, achieving an ultra-high PCF, and demonstrating great potential for precise pressure measurement applications. To further enhance the temperature stability of the sensor, in addition to employing an external circuit temperature compensation method and introducing a temperature compensation layer, Tao Wang et al. [23] designed a sensor with a dual temperature compensation structure (as shown in Figure 13c). They deposited SiO_2_ on the top of the sensor as a temperature compensation layer to improve its temperature characteristics and integrated a vacuum chamber to achieve low-temperature drift by reducing the impact of environmental pressure on the sensor. This design ensures that the sensor maintains high-precision pressure measurement even in environments with significant temperature variations. Future research can further optimize the material selection and structural design of sensors, as well as explore higher performance temperature compensation methods to meet continuously changing application demands.

### 4.4. LWR Humidity Sensors

Humidity sensors typically employ hygroscopic materials as the sensing layer. To enhance the sensitivity of sensors, current research often utilizes nanomaterials as the sensing layer due to their larger specific surface area and high surface activity [105]. The humidity sensor based on the LWR primarily utilizes the two-dimensional nanomaterial, mainly graphene oxide (GO) as the sensing layer. The carbon plane of GO contains numerous oxygen-containing groups, facilitating the adsorption of water molecules, and its excellent electrical properties enable the sensor to respond rapidly to changes in humidity. Additionally, materials such as PVA/graphene flower [106], Cd-ZnO nanowires [107], and CuO nanosheets [108] can also function as sensing layers for humidity sensors, each offering unique physical and chemical properties that provide varying sensitivities and response times. Therefore, future developments may explore other materials to further expand the performance and application range of LWR humidity sensors.

The Lamb wave humidity sensor detects changes in environmental humidity through the propagation of the Lamb wave on the piezoelectric thin plate. A layer of GO thin film is coated on the piezoelectric thin plate of the sensor. When water molecules in the air are adsorbed by the GO thin film, their presence alters the mass of the resonator. Since there is a linear relationship between the mass load on the surface of the resonator and the frequency offset, this phenomenon can be explained by the Sauerbrey equation, as follows [109]:

(22) $$∆f=-Cf_{0}^{2}∆m/A$$

where C is a constant related to the piezoelectric thin plate, $f_{0}$ is the natural resonant frequency of the humidity sensor, and Δm/A is the mass change per unit area.

In fact, the thickness of the GO sensing layer has a significant impact on the sensitivity of the sensor. A thicker GO sensing layer can offer more oxygen-containing groups for adsorbing water molecules, thereby enhancing the sensitivity of the sensor. However, increasing the thickness also leads to a greater mass load, which, in turn, causes frequency hysteresis. The sensitivity of the humidity sensor is defined as follows [110]:

(23) $$S_{m}=\frac{∆f}{∆RH}$$

where ΔRH is the change in relative humidity, and Δf is the change in resonant frequency caused by the relative humidity.

The maximum relative frequency hysteresis H is defined as the ratio of the maximum absolute frequency hysteresis ($∆f_{max}$) to the sensor sensitivity at the point of maximum absolute frequency hysteresis ($S_{0}$), as follows [20]:

(24) $$H=\frac{∆f_{max}}{S_{0}}$$

**Figure 14:**
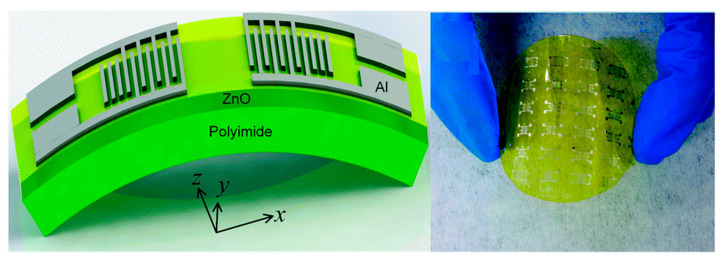
Structural and physical diagram of a flexible dual-mode (A_0_ and S_0_) LWR humidity sensor [19].

Recently, researchers have proposed some methods and structures to obtain high sensitivity and low hysteresis humidity sensors. Weipeng Xuan et al. [19] designed a flexible Lamb wave humidity sensor that combines A_0_ and S_0_ modes, with its 3D structural diagram and physical image shown in Figure 14. The device utilizes polyimide tape as a flexible substrate and experimentally compares the performance differences of the sensor with and without a graphene oxide (GO) sensing layer. The results indicate that the GO sensing layer can significantly enhance sensitivity. Additionally, the study demonstrated that this flexible sensor could operate normally even under severe bending conditions, making it suitable for flexible wearable devices and other applications. Jintao Pang et al. [110] designed humidity sensors coated with GO thin films at different concentrations and proposed a vacuum deposition method to improve the performance of the GO thin film. Experimental results showed that GO concentration significantly affects the frequency hysteresis of the sensor. Moreover, the sensor exhibited a low temperature coefficient, low hysteresis, and short response/recovery times, demonstrating good application potential. Unlike the aforementioned studies, Amardeep Singh Dhillon et al. [21] did not use GO as the sensing layer but instead employed ZnO directly as the sensing medium. To reduce the impact of acoustic wave damping in the flexible device, SU-8 was used as a polymer interlayer material to reduce the damping effect caused by film resonance. This design effectively improved the performance of the sensor, resulting in higher sensitivity and stability in flexible humidity sensing applications. Table 6 summarizes several characteristics of the Lamb wave humidity sensors reported by researchers.

## 5. Conclusions

Lamb wave sensors have significant advantages in sensing technology. Compared to SAW and BAW sensors, Lamb wave sensors exhibit higher phase velocities, larger electromechanical coupling coefficients, and lower dispersion, making them more sensitive to external changes and capable of detecting smaller variations. To enhance the quality factor and effective electromechanical coupling coefficient of LWRs and thereby improve the sensitivity of Lamb wave sensors, electrode configurations can be optimized, such as by adopting a double-IDT electrode configuration. Additionally, fabrication processes can be improved, such as by using butterfly-shaped thin plates and chamfered corner thin plates in structural design to reduce anchor loss and the impact of parasitic modes.

Lamb wave sensors have extensive applications in various fields. In biosensing, they exhibit high sensitivity in detecting target molecules such as DNA, bacteria, and biopharmaceuticals. In liquid sensing, Lamb wave sensors can be employed to measure parameters such as viscosity, density, and moisture content. In pressure sensing, Lamb wave sensors demonstrate excellent performance in effectively detecting external pressure changes. Additionally, humidity sensors are used to measure the humidity in air or materials. To further enhance the performance of these sensors, the thickness of the piezoelectric material can be adjusted, appropriate electrode materials can be selected, and electrode configurations can be optimized. For example, when LWRs are used as liquid sensors, employing Mo with high Young’s modulus and excellent corrosion resistance or Au/Ti with high conductivity and stability as electrodes can improve the device’s stability in liquid environments, thereby achieving better sensing performance.

Currently, the application of Lamb wave sensors in integrated circuits and microsystems still faces several challenges, necessitating further improvements in sensor sensitivity and overcoming fabrication process challenges. Future development trends include the adoption of novel materials and structures to enhance sensor performance and the exploration of additional applications. Biosensors will continue to optimize sensitivity and detection limits, liquid sensors will focus on improving durability and environmental adaptability, and pressure sensors will aim to enhance stability and accuracy. Through these improvements and developments, Lamb wave sensors are expected to play an increasingly significant role across various fields of modern technology.

## Figures and Tables

**Table 1:** Piezoelectric materials used in the development of Lamb wave sensors [67,68,69,70,71,72,73,74,75,76,77,78,79].

Material	LiNbO_3_	AlN	ZnO	GaN
Crystal structure	Trigonal	Wurtzite	Wurtzite	Wurtzite
Bandgap/eV	3.95	6.3	3.35	3.39
$\mathrm{Longitudinal}\;\mathrm{wave}\;\mathrm{acoustic}\;\mathrm{velocity}/m\cdot s^{-1}$	6500–7365	10,150–11,050	6340	8040
$\mathrm{Shear}\;\mathrm{wave}\;\mathrm{acoustic}\;\mathrm{velocity}/m\cdot s^{-1}$	-	5800	2700–2720	4130
$\mathrm{Electron}\;\mathrm{mobility}/{\mathrm{cm}}^{2}V^{-1}s^{-1}$	0.8	135	200	1000
$\mathrm{Density}/g\cdot {\mathrm{cm}}^{-3}$	4.64	5.61–5.72	3.25–3.3	6.095–6.15
$\mathrm{Dielectric}\;\mathrm{constant}/(10^{-11}F\cdot m^{-1}$)	85(29)	8.5	8.66	8.9
$\mathrm{Effective}\;\mathrm{electromechanical}\;\mathrm{coupling}\;\mathrm{coefficient}\;k_{eff}^{2}$(%)	5–11.3	3.1–8	1.5–1.7	0.13
$\mathrm{Acoustic}\;\mathrm{impedance}\;(10^{6}\;\mathrm{kg}\cdot (m^{-2}s^{-1})$)	35	36.5	35.6	45

**Table 3:** Performance comparison of fabricated biosensors.

Piezoelectric Material	Sensor Structure	Wave Mode	Operating Frequency/MHz	Detection Target	Sensitivity	Detection Limits/Pgul^−1^	Reference
LiNbO_3_	PMMA/Mo/LN/SiO_2_/Si	S_1_	8000	Biomolecule	$74,000\;(\mathrm{Hz}/\left(\mathrm{ng}/{\mathrm{cm}}^{2}\right)$)	-	[12]
ZnO	ZnO/SiO_2_/Si/SiO_2_/ZnO/Al	A_0_, S_0_	91.55~137.44	DNA	$202–310\;(\mathrm{Hz}/\left(\mathrm{ng}/\mathrm{nl}\right)$)	82~84	[13]
ZnO	Cr/Au/ZnO/Al	A_0_, A-TSM, S-TSM	7.41~14.1	Imatinib	$1.1\;(\mathrm{kHz}/{\mathrm{cm}}^{2}\cdot \mu M$)	-	[14]
AlN	PMMA/Ni/AlN/SiO_2_	A_0_	129.96~139.7	Biomolecule	$34.5–63.9\;(\mathrm{Hz}/\left(\mathrm{ng}\cdot {\mathrm{cm}}^{2}\right)$)	-	[95]

**Table 4:** Performance comparison of fabricated liquid sensors.

Piezoelectric Material	Sensor Structure	Operating Frequency/MHz	Detection Target	Q Factor	Sensitivity	Reference
AlN	Al/AlN/Mo/Si	851~881	Dielectric permittivity, Viscosity, Density	1200~1400	-	[17,18,98]
GaN	Cr/Au/GaN/seed layer/Si	142~458	Viscosity, Protein-antibody	-	$6.4\sim 7.1\;({\mathrm{cm}}^{2}/\left(\mathrm{gr}\right)$)	[103]
AlN	Al/AlN/SOI	-	Viscosity Density	-	η$:-569\;(\mathrm{ppm}\cdot \mathrm{mPa}\cdot s^{-1}$) ρ$:-748\;(\mathrm{ppm}\cdot g^{-1}\cdot {\mathrm{cm}}^{-3}$)	[16]
AlN	Au/Ti/AlN/SOI	327.7	Viscosity	-	-	[15]

**Table 5:** Performance comparison of fabricated pressure sensors.

Piezoelectric Material	Sensor Structure	Wave Mode	Temperature Range/°C	Operating Frequency /MHz	$\mathrm{TCF}1/\mathrm{ppm}\cdot {℃}^{-1}$	$\mathrm{TCF}2/\mathrm{ppb}\cdot {℃}^{-2}$	$\mathrm{PCF}/\mathrm{ppm}\cdot {\mathrm{psi}}^{-1}$	Reference
AlN	Al/AlN/Si/SiO_2_/Si	LFE	−50~300	481.4	−19.03	−13.17	−10.28	[46]
AlN	Al/AlN/SiO_2_/SoI	LFE, SAW	−50~300	478~988	−21.14~−21.49	−21.70~−23.53	−0.612~+0.227	[22]
LiNbO_3_	Cr/Au/LN/SiO_2_/Si/Glue/Si	SH_0_, SH_1_, SH_2_, A_1_	−40~100	424.2~885.4	−3.18~−26.57	-	6.21~41.38	[104]
AlN	Mo/AlN/Mo/AlN/SOI	-	20~220	819.5	−14.4	-	18.28	[23]

**Table 6:** Performance comparison of fabricated humidity sensors.

Piezoelectric Material	Sensor Structure	Wave Mode	Operating Frequency /MHz	Sensitivity	$\mathrm{TCF}/\mathrm{ppm}\cdot {℃}^{-1}$	Frequency Hysteresis/kHz	Reference
ZnO	GO/Al/ZnO/PI	A_0_, S_0_	150~395	25.72–145.83 (ppm/85%RH)	-	-	[19]
AlN	GO/Cr/Al/PSG/AlN/SOI	A_0_	~390	26 (kHz/%RH)	−27.2~−28.9	12~20	[110]
ZnO	Al/ZnO/Al/SU-8/Si/SU-8	S_0_	65.82	~105 (kHz/50%RH)	-	-	[21]

## Data Availability

The raw data supporting the conclusions of this article will be made available by the authors on request.
